# IL-17-producing ^©^™T cells are important for the development of arthritis in a rheumatoid arthritis model

**DOI:** 10.1186/ar3604

**Published:** 2012-02-09

**Authors:** Aoi Akitsu, Harumichi Ishigame, Shigeru Kakuta, Shinobu Saijo, Yoichiro Iwakura

**Affiliations:** 1 Center for Experimental Medicine and Systems Biology, The Institute of Medical Science, The University of Tokyo, Japan; 2 Japan Society for the Promotion of Science (JSPS) 3Core Research for Evolutional Science and Technology (CREST), Japan

## Background

IL-1 receptor antagonist deficient (*Il1rn*-/-) mice spontaneously develop arthritis. We previously demonstrated that IL-17 plays a crucial role in the development of arthritis in *Il1rn*-/- mice. Furthermore we showed that IL-1 Ra-deficiency in T cells is important for the development of arthritis. It is not known, however, which IL-17-producing cells are involved in the pathogenesis of arthritis in this model.

## Results

To identify the source of IL-17 in *Il1rn*-/- mice, we analyzed IL-17-producing cells. We found that IL-17 production from both CD4+ T cells and ^©^™T cells was increased in the draining lymph nodes. To clarify the roles of CD4+ T cells and ^©^™T cells in the development of arthritis, ^©^™T cells or CD4+ T cells were depleted in *Il1rn*-/- mice using antibodies. The development of disease was suppressed in both cases, suggesting both Th17 cells and IL-17-producing ^©^™T cells were involved in the pathogenesis. Then, the pathogenic role of IL-17-producing ^©^™T cells in the absence of Th17 cells was examined.

We generated mice with IL-17 producing ^©^™T cells, but without Th17 cells, by adoptively transferring *Il17-/-Il1rn*-/--T cells into nude mice in which IL-17-producing ^©^™T cells are present. We found that these mice still developed arthritis and that only ^©^™T cells produced IL-17. Finally, to corroborate that the development of arthritis in this transfer system is dependent on IL-17, we adoptively transferred *Il17-/-Il1rn*-/-**-**T cells into *Il17-/-nu/nu *mice. The development of arthritis was significantly suppressed in *Il17-/-Il1rn*-/-**-**T cell-transferred *Il17-/-nu/nu *mice compared with *Il-17+/+nu/nu *mice transferred with *Il17-/-Il1rn*-/-**-**T cells, suggesting that ^©^™T -cell-derived IL-17 is important for the develop arthritis.

## Conclusion

These results indicate that ^©^™T cell-derived IL-17 plays an important role in the pathogenesis of arthritis in *Il1rn*-/- mice.

